# Meta-analysis of tumour burden in pre-operative axillary ultrasound positive and negative breast cancer patients

**DOI:** 10.1007/s10549-017-4405-3

**Published:** 2017-07-28

**Authors:** Muneer Ahmed, F. Jozsa, R. Baker, I. T. Rubio, J. Benson, M. Douek

**Affiliations:** 1 Division of Cancer Studies, Research Oncology, King’s College London, Guy’s Hospital Campus, Great Maze Pond, London, SE1 9RT UK; 2 Department of Statistics, School of Business, University of Salford, 612, Maxwell Building, Salford, M5 4WT UK; 3 Breast Surgical Unit, Breast Cancer Centre, Hospital Universitario Vall d’Hebron 119-129, 08035 Barcelona, Spain; 4 Breast Surgical Unit, Addenbrooke’s Hospital, Cambridge, CB2 0QQ UK

**Keywords:** Breast cancer, Axillary ultrasound, Sentinel lymph node biopsy, Axillary node clearance, Axillary burden

## Abstract

**Background:**

Management of the axilla in breast cancer is becoming increasingly conservative. Patients identified with a low axillary nodal burden (two or fewer involved nodes) at sentinel node biopsy (SNB) can avoid completion axillary node clearance (cANC). ‘Fast track’ to ANC in patients with involved nodes on pre-operative ultrasound may be over-treating a subgroup of these patients with low nodal burden, which would have precluded their need for ANC. This systematic review assesses the proportion of patients with involved nodes on pre-operative axillary ultrasound, which would fit low axillary burden criteria.

**Methods:**

Meta-analysis of studies comparing axillary burden of breast cancer patients identified as pre-operative ultrasound negative versus positive was performed. The primary outcome measure was the number of patients with two or fewer involved nodes (macrometastases only). Pooled odds ratio (OR), 95% confidence intervals (CIs), means and probabilities of identifying two or fewer involved nodes versus greater than two were calculated.

**Results:**

Six studies reported the axillary burden in 4271 patients who were either directed straight to ANC or cANC after SNB. There was a significantly greater axillary burden in the ultrasound positive versus negative groups (OR 5.95, 95% CI 5.80–6.11) with mean nodal retrieval values of 2.9 [standard error (SE) 0.2] and 1.6 (SE 0.2) nodes, respectively. Cumulative probabilities identified 78.9% of ultrasound negative and 43.2% of ultrasound positive patients possessed low axillary burden.

**Conclusions:**

Pre-operative ultrasound positive patients have significantly higher axillary burden. However, nearly half do fit the criteria of low axillary burden and could be considered for omission of ANC.

## Introduction

The management of the axilla in breast cancer has been increasingly progressing towards a more conservative approach. This commenced with the omission of completion axillary node clearance (cANC) for isolated tumour cells (ITCs) [1], before progressing to omitting it for micrometastases [2] and then for low burden axillary macrometastatic disease [3, 4] identified at sentinel node biopsy (SNB)—in the presence of systemic therapy or radiotherapy [3]. Pre-operative axillary ultrasound (with core biopsy or fine needle aspiration) has become a standard to stratify patients with high burden axillary disease directly towards axillary clearance (ANC) and omission of SNB, despite the fact that axillary ultrasound was not required in any of the large randomized controlled trials (RCTs) of SNB [5–10]. Pre-operative axillary ultrasound has traditionally not been able to replace SNB because of its inferior sensitivity compared to the latter [11, 12]. Therefore, the identification of axillary disease on ultrasound is considered a prognostic indicator of higher axillary burden requiring ‘fast track’ to ANC and omission of SNB [13]. However, the lack of clear criteria to stage the axilla and inter-personal variation in the performance of ultrasound means that there potentially is a risk in the modern era of overtreatment of the axilla in a certain group of patients who may have fitted the category of low axillary burden (two or fewer involved nodes with macrometastases) on SNB. The aim of this meta-analysis is to determine the proportion of patients directed to ‘fast track’ ANC who are found to subsequently possess low axillary burden on histopathology and consequently could have potentially avoided ANC.

## Methods

### Study selection

A systematic review of the literature was performed using MEDLINE, PubMed, Embase and Cochrane Library databases to identify all articles evaluating the histopathology of axillary specimens for metastatic nodal burden after ANC in breast cancer. The search terms used were: SNB and axillary ultrasound and axillary lymph node clearance and axillary lymph node dissection and breast cancer. Studies were restricted to those published in the English language and performed in humans. The related articles function was used to broaden the search, and all abstracts, studies and citations obtained were reviewed. References of the acquired articles were also searched by hand. The last search was conducted on 1st February 2017.

### Inclusion criteria

Studies were included if they satisfied the following eligibility criteria: performance of a comparison of the histological metastatic axillary nodal burden at ANC between breast cancer patients ‘fast tracked’ to ANC [due to a positive pre-operative axillary ultrasound scan (US) with core biopsy or fine needle aspiration] and those with clinically and radiologically negative axillae who were directed to SNB before undergoing cANC for macrometastatic disease; quantified the resultant axillary nodal burden at ANC in terms of 0–2 nodes and greater than 2 nodes (‘low’ and ‘high’ axillary burden, respectively); a satisfactory quality assessment score attained (4 of 6 or greater) and written in the English language.

### Exclusion criteria

Studies that failed to fulfil the inclusion criteria or studies in which the outcomes of interest were not reported were excluded. Other exclusion criteria included: full text not available, review article, letter to the editor, editorial report, case report, duplicate publication, abstract.

### Data extraction

Data were extracted from selected studies using a data extraction form, which included information on: publication details; study design; number of patients; number of axillary US positive patients—with positive core biopsy or fine needle aspiration—and their nodal burden at ANC; number of axillary US negative and SNB positive patients and their nodal burden at ANC. The quality of cohort studies was assessed according to the recommendations of the Strengthening the Reporting of Observational studies in Epidemiology (STROBE) statement [14] and six items of the STROBE statement were considered relevant for quality evaluation. Two-reviewers extracted data from included studies independently. Comparison of the data extraction and quality score was performed, and discrepancies were resolved by consensus.

### Statistical analysis

All extracted data were tabulated and presented numerically in terms of patient numbers according to pre-operative axillary status and their subsequent allocation to ‘low’ (fewer than two macrometastases) or ‘high’ (greater than two macrometastatic nodes) axillary burden. The Mantel–Haenszel test (Cochran) [15] was used to determine the difference in axillary burden between the pre-operative US positive and negative groups. The odds ratios (ORs) and 95% confidence intervals (CIs) were calculated. Means and probabilities of axillary nodal burden were calculated by fitting a beta-binomial distribution to the number of involved nodes using the method of maximum likelihood.

## Results

**Fig. 1:**
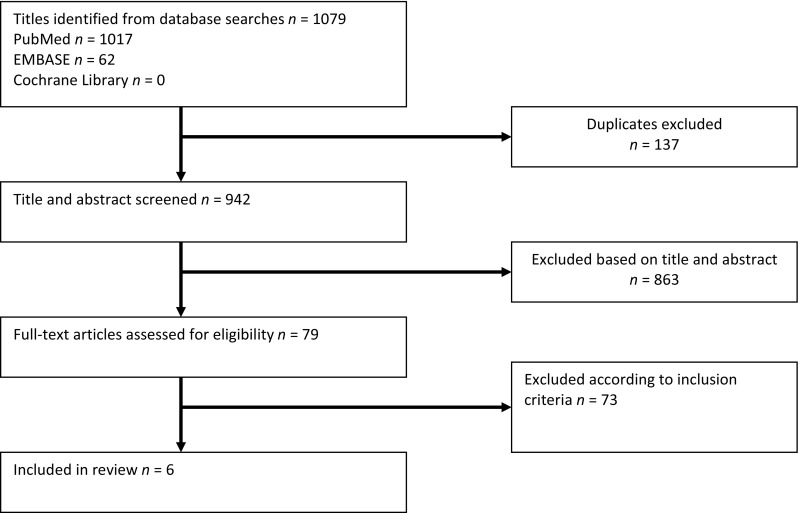
Selection of articles for review

The detailed literature search resulted in six studies being critically appraised for this review (Fig. 1) [16–21].

### Study characteristics

The studies were published between 2014 and 2016. They comprised six cohort studies [16–21] of which all were retrospectively conducted—three using prospectively collected databases [17–19].

### Study quality

The relevant items of the STROBE statement that were used for quality assessment of included cohort studies are shown (Table 1). The overall STROBE score ranged from 4 to 6. The methodology and reported data of all included cohort studies were considered adequate.

**Table 1:** Study quality assessment of cohort studies

References	Study objectives	Clear inclusion criteria	Standardized technique	Only proven malignancy	Patient follow-up reported	Withdrawals from study reported
Barco et al. [16]	Y	Y	Y	Y	N	N
Boland et al. [17]	Y	Y	Y	Y	N	Y
Boone et al. [18]	Y	Y	Y	Y	N	N
Moorman et al. [20]	Y	Y	Y	Y	N	N
Caudle et al. [19]	Y	Y	Y	Y	N	N
Verheuvel et al. [21]	Y	Y	Y	Y	Y	N

Study quality was assessed according to the Strengthening the Reporting of Observational Studies in Epidemiology (STROBE) statement

*Y* yes, *N* no

### Study outcomes

#### Clinically significant axillary burden

**Fig. 2:**
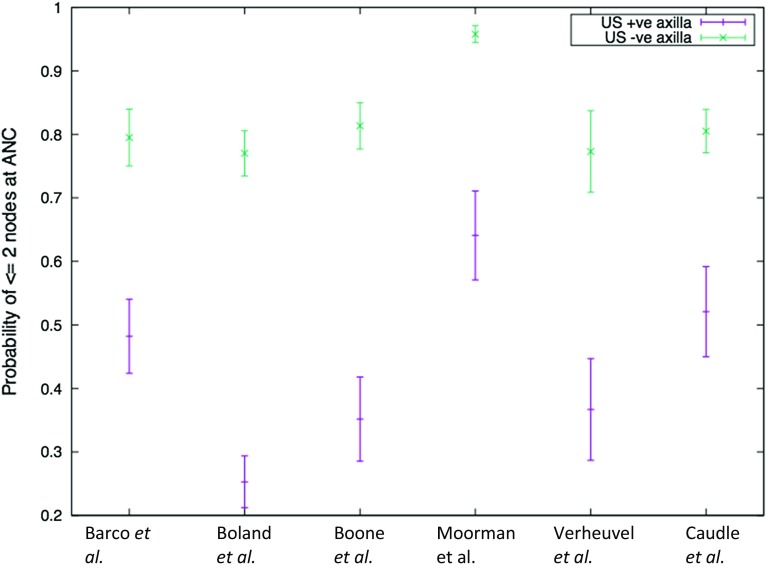
The 95% confidence intervals for the probabilities of identification of two or fewer involved nodes on pre-operative ultrasound negative and positive patients

**Fig. 3:**
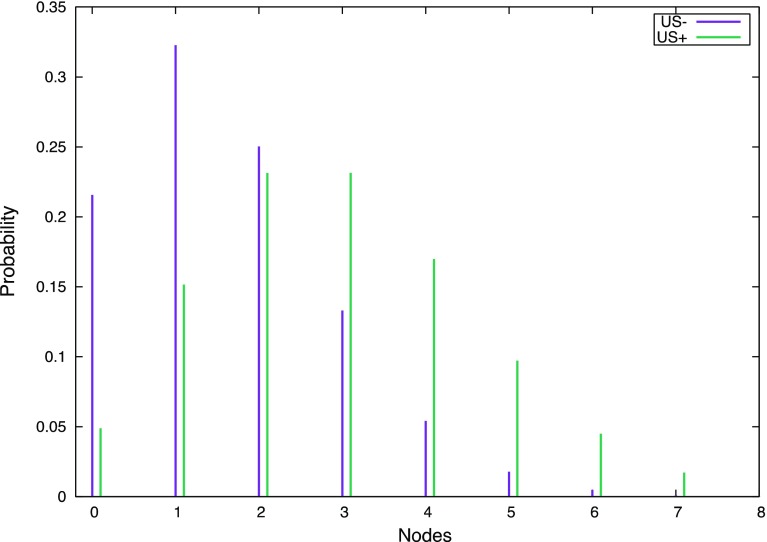
Estimated axillary metastatic nodal distribution according to pre-operative ultrasound status from binomial expansion of recorded data

Six studies [16–21] comprising 4271 patients provided data on the presence of ‘high’ versus ‘low’ axillary burden—on histology at ANC—in patients ‘fast tracked’ to ANC after positive axillary scanning (1437 patients) and who underwent cANC after positive SNB (greater than two macrometastases; Table 2). There was significantly greater ‘high axillary burden’ identified in the ultrasound positive versus negative group (OR 5.95, 95% CI 5.80–6.11) and is graphically represented by the 95% CIs for the probabilities of two or fewer involved nodes in these two groups (Fig. 2). The beta-binomial model provided a mean number of macrometastatically involved nodes excised at ANC of 2.9 [standard error (SE) 0.2] and 1.6 (SE 0.2) for the US positive and negative groups, respectively. The beta-binomial fitted distribution of expected involved nodes (Fig. 3) for the two groups demonstrates the mode at one node for the US negative (SNB positive) and two to three nodes for the US positive group. The cumulative probabilities identify that 78.9% of US negative patients have two or fewer involved nodes compared to 43.2% of US positive patients.

**Table 2:** Study characteristics and outcomes of axillary nodal burden in pre-operative ultrasound positive and negative groups

References	Study type	Total number of patients	Pre-operative US positive axilla			Pre-operative US negative axilla (SNB positive)		
			Number of patients	≤2 Nodes at ANC	>2 Nodes at ANC	Patient numbers	≤2 Nodes at ANC^b^	>2 Nodes at ANC^b^
Barco et al. [16]	Retrospective cohort	594^a^	282	136	146	312	248	64
Boland et al. [17]	Retrospective cohort	974	439	112	327	535	412	123
Boone et al. [18]	Retrospective cohort	633	199	70	129	434	353	81
Moorman et al. [20]	Retrospective cohort	1060	181	116	65	879	842	37
Verheuvel et al. [21]	Retrospective cohort	302	139	51	88	163	126	37
Caudle et al. [19]	Retrospective cohort	708	190 (149)	99 (82)	91 (67)	518	417	101

( ) Proportion of patients with one or two suspicious lymph nodes on ultrasound

^a^Selection of patient numbers according to those identified as node positive from the data

^b^Cumulative number of involved nodes identified—at sentinel node biopsy and completion axillary clearance

Only one study [19] performed a quantification of the number of suspicious nodes identified on axillary ultrasound. Caudle et al. [19] identified that 78% of pre-operative ultrasound positive patients had one or two suspicious nodes identified. Of these patients 55% had two or fewer involved nodes on histopathology at ANC. They were the only authors to perform multivariate analysis of predictors of high axillary burden within the ultrasound positive group. They identified that there was a significant relationship between lobular histology and high axillary burden (three or more involved nodes, OR 1.77; 95% CI 1.06–2.95) at ANC.

## Discussion

The management of the axilla in breast cancer has seen the emergence of surgical conservatism away from ANC in view of its lack of disease-free and overall survival benefit [4, 22, 23]. This has resulted in the increasing importance of the quantification of axillary disease in order to differentiate between ‘high’ and ‘low’ axillary burden. The current criteria for this is based upon those established within the ACOSOG Z0011 trial [4] and then formally supported by the American Society of Clinical Oncologists (ASCO) [24]. During the ACOSOG Z0011 [4] trial pre-operative axillary ultrasound was not performed. This is directly in conflict with mainstream practice, which directs those patients with suspicious axillary findings on ultrasound to undergo tissue sampling by either core biopsy or fine needle aspiration. Those patients identified to have metastatic disease pre-operatively, are streamlined directly to ANC. This means that out of those patients who have metastatic axillary disease, one-third (Table 2) of them would have undergone SNB rather than ‘fast track’ ANC if they were part of the ACOSOG Z0011 trial and half of these would have fit criteria of ‘low axillary burden’ and been omitted ANC. This certainly is suggestive that the use of pre-operative ultrasound is directing a greater number of patients to ANC and denying them axillary preservation than is otherwise necessary.

It has been established that axillary burden in those patients with pre-operative positive ultrasound findings is greater than in those that are negative. Van Wely et al. [13] in their meta-analysis of ultrasound-guided biopsy for determining axillary burden demonstrated that the number of involved nodes was significantly higher in patients in whom axillary metastases were identified by ultrasound-guided biopsy (*P* < 0.001). Unfortunately, due to the limitations of the data available at the time, the authors were only able to categorize axillary burden in the US positive and negative groups to one to three and greater than three nodes and were unable to segregate ITC, micro and macrometastatic disease. This prevented the relevance of their data to modern guidelines and the criteria for ‘low and high axillary burden’ [24]. This meta-analysis supports the finding of van Wely et al. [13] as we too found that there was significantly higher axillary burden in the pre-operative positive US group compared to the negative one (OR 5.95, 95% CI 5.80–6.11). This was at the clinically relevant threshold of two nodes or fewer and greater than two nodes, in addition to only considering macrometastases. Our meta-analysis also demonstrated that the mean number of nodes expected to be retrieved in patients with a positive US is twice that of those with a negative US [3.0 vs. 1.6 (SE 0.2)]. It has also been shown that the mode distribution of involved nodes is one and two to three for US negative and positive patients, respectively. This clearly demonstrates that axillary burden is higher in the US positive group. It is logical, as the sensitivity of US has been consistently shown to be inferior to that of SNB for the identification of metastatic disease [11, 12]. Therefore, for disease to be visible to the radiologist one would expect that it might be of greater quantity.

However, in an era of increasingly targeted and personalized therapy, it is not adequate to consider these data purely within the context of ‘greater axillary burden’ present in the US positive group. This meta-analysis identified 43% of US positive patients had two or fewer nodes consistent with low axillary burden, which would allow them to avoid ANC. This raises the question of how do we prevent the overtreatment of this group? For the 43% of patients receiving an unnecessary ANC, a possibility could exist in avoiding the performance of pre-operative axillary ultrasound. Such a technique would increase the patients directed to SNB rather than ‘fast track’ ANC by approximately one-third and of this one-third, half would avoid cANC on the basis of subsequent SNB histology. The only patients fast tracked to ANC would be those patients with palpable axillary lymphadenopathy—consistent with the protocols of RCTs of SNB [5–10]. This approach would make a contribution, but may give the appearance of a regressive step and could be detrimental in patients with a high body mass index where axillary ultrasound has been demonstrated to possess great accuracy [25]. It is known that this approach to omitting pre-operative axillary ultrasound has already been adopted in certain academic breast units within the USA. However, published data on the outcomes of this practice are not currently available and the American Society of Breast Surgeons [26] still advocates pre-operative axillary ultrasound in the presence of diagnosed breast cancer. Another approach would be to improve quantification of axillary US findings—which is difficult with the inter-personal variation associated with the performance of US. However, the impetus should be placed upon the radiologist to quantify the number of suspicious nodes visible. The suitable threshold to be used for this would have to be ascertained through further clinical evaluation, as it cannot be assumed to extrapolate to two or fewer visible nodes on US. Only the study by Caudle et al. [19] demonstrated that by using a threshold of two suspicious nodes or fewer that the number of patients with two or fewer involved nodes at ANC was 55%. If a threshold of one suspicious node only was applied this percentage would inevitably increase. Therefore, it is likely that the cut off on axillary ultrasound should be one to two suspicious nodes but confirmation of this should form the basis of future research—as such data are minimal within the literature. In addition to quantification, the suspicious nodes could also be marked pre-operatively at US using clips, which could then allow targeted intra-operative excision. This technique has successfully reduced the false negative rate at SNB within the neo-adjuvant chemotherapy setting to under 2% [27]. It has to be stressed that unless ultrasound evolves and is approached in this manner, it is likely to become disadvantageous and its role increasingly questionable.

Axillary radiotherapy could play a role in management but it must be remembered that the AMAROS trial [3] did not use pre-operative axillary ultrasound and excluded patients on the basis of palpable lymphadenopathy—consistent with the original trials of SNB [5–10] and ACOSOG Z0011 [23]. Whilst there was no threshold limit of axillary burden to exclude patients from randomization, only 5% of patients in the radiotherapy and ANC arms had a burden of three or more involved nodes at SNB. It is also important to note that only 60% of involved nodal burden in each arm consisted of macrometastases. Axillary radiotherapy certainly could be considered a therapeutic option for the 43% of patients who fit the low axillary burden criteria (two or fewer involved nodes) despite a histologically involved axilla on pre-operative US. However, as a blanket treatment of patients with burden greater than two nodes, it would be unproven.

The issue of patients undergoing mastectomy and their axillary burden was not addressed by the ACOSOG Z0011 trial [23]. Within the included trials of this meta-analysis, mastectomy rates varied between 32 and 65% [19, 21]. Whilst it may be assumed that the ACOSOG Z0011 criteria could be extrapolated to mastectomy patients, it would mean that patients who would have avoided chest wall radiotherapy in the event of ANC may become committed to it if no further intervention is performed to the axilla. This would be the only safe option in the absence of further clinical trial data.

It is also acknowledged that whilst attempting to avoid ANC in the identified cohort of 43%, it means that the remaining 57% of patients, who do not fit the criteria of ‘low axillary burden’ will have to undergo two operations with the associated additional exposure to a general anaesthetic and potential financial implications—although the latter could be offset by the saving in the ‘low axillary burden’ group avoiding ANC. In other aspects such as the administration of primary chemotherapy, this approach would not detrimentally influence patient management and would allow accurate axillary staging with SNB post-chemotherapy, especially if ‘clipping of suspicious nodes’ is performed [27, 28].

The use of other imaging modalities should be considered to complement physical examination, particularly in obese patients where such clinical assessment is very challenging. Magnetic resonance imaging (MRI) has failed in mainstream uptake for axillary staging in breast cancer due to its high false positive rate when compared to SNB (6.3 vs. 0.2%, respectively) [29]. This is a significant limitation when a key aim of modern axillary management is to avoid overtreatment of the axilla or if MRI is being used to identify ‘high axillary burden’ and ‘fast track’ to ANC—avoiding SNB altogether. A systematic review of positron emission tomography (PET) for the assessment of axillary lymph node status in early breast cancer found that across 26 studies evaluating PET or PET/CT, the mean sensitivity and specificity was 63 and 94%, respectively [30]. Clearly, these areas of research should continue to be explored but will also face challenges within the financial constraints of public healthcare systems.

It must be remembered that such advancements are not only targeted at reducing the 43% of patients with positive US who have an unnecessary ANC but also the 79% of US negative patients with low axillary burden found on SNB who could potentially have avoided SNB. The case of the latter may well be addressed by ongoing current research [31] but the 43% provide a greater long-term area of concern. Only the article by Caudle et al. [19] attempted to identify discriminating factors for high axillary burden within the ultrasound positive group. On multivariate analysis they identified lobular histology as a risk factor (OR 1.77; 95% CI 1.06–2.95). Urgent analysis of further risk factors within this group, are warranted to prevent overtreatment and could potentially involve the use of molecular profiling. In the void of advanced imaging modalities, quantification of axillary ultrasound is essential in non-palpable axillary disease. Within this meta-analysis, none of the included articles [16–21] provided any details on the quantification of axillary disease on US, highlighting that this is clearly an area, which needs to be improved upon. It would be suggested that the number of abnormal axillary lymph nodes should be clearly stated by the conducting radiologist. Those with inconsistent ultrasound findings or a single abnormal node should be offered SNB without axillary ultrasound directed biopsy. Those patients with more than one abnormal node or those with an abnormal node who are about to commence primary chemotherapy, should be considered for biopsy and clipping of the node(s). Further studies are needed to refine the technique of clipping the node and SNB with removal of the clipped node. The only other current option would be to avoid axillary ultrasound altogether to avoid overtreatment.
